# Psychological Health and Wellness and the Impact of a Supportive Text Messaging Program (Wellness4MDs) Among Physicians and Medical Learners in Canada: Protocol for a Longitudinal Study

**DOI:** 10.2196/44368

**Published:** 2024-09-16

**Authors:** Reham Shalaby, Belinda Agyapong, Raquel Dias, Gloria Obuobi-Donkor, Medard K Adu, Sharron Spicer, Natalie L Yanchar, Vincent I O Agyapong

**Affiliations:** 1 Department of Psychiatry University of Alberta Edmonton, AB Canada; 2 Department of Psychiatry Faculty of Medicine Dalhousie University Halifax, NS Canada; 3 Office of the Chief Medical Officer Alberta Health Services Calgary, AB Canada; 4 Department of Surgery Cumming School of Medicine University of Calgary Calgary, AB Canada

**Keywords:** wellness, doctors, Canada, depression, burnout, anxiety, supportive text messages, eHealth

## Abstract

**Background:**

Burnout, anxiety, and depression continue to affect physicians, postgraduate medical trainees, and medical students globally and in Canada particularly after the COVID-19 pandemic.

**Objective:**

The primary goal of this project is to design, implement, monitor, and evaluate a daily supportive SMS text messaging program (Wellness4MDs, Global Psychological e-Health Foundation). The program aims to reduce the prevalence and severity of burnout, anxiety, and depression symptoms among physicians, postgraduate medical trainees, and medical students in Canada.

**Methods:**

This longitudinal study represents a multistakeholder, mixed methods, multiyear implementation science project. Project evaluation will be conducted through a quantitative prospective longitudinal approach using a paired sample comparison, a naturalistic cross-sectional controlled design, and satisfaction surveys. Prevalence estimates for psychological problems would be based on baseline data from self-completed validated rating scales. Additional data will be collected at designated time points for paired comparison. Outcome measures will be assessed using standardized rating scales, including the Maslach Burnout Inventory for burnout symptoms, the 9-item Patient Health Questionnaire for depression symptoms, the 7-item Generalized Anxiety Disorder scale for anxiety symptoms, and the World Health Organization–Five Well-Being Index.

**Results:**

The project launched in the last quarter of 2023, and program evaluation results will become available within 36 months. The Wellness4MDs program is expected to reduce the prevalence and severity of psychological problems among physicians in Canada and achieve high subscriber satisfaction.

**Conclusions:**

The results from the Wellness4MDs project evaluation will provide key information regarding the effectiveness of daily supportive SMS text messages and links to mental health resources on these mental health parameters in Canadian physicians, postgraduate trainees, and medical students. Information will be useful for informing policy and decision-making concerning psychological interventions for physicians in Canada.

**International Registered Report Identifier (IRRID):**

PRR1-10.2196/44368

## Introduction

Burnout, anxiety, and depression continue to be a problem among physicians globally and in Canada. The recent Canadian Medical Association National Physician Health Survey shows that the well-being of physicians across Canada has significantly decreased [1]. The survey demonstrates that many physicians report poorer mental health than before the COVID-19 pandemic, with 53% of respondents nationally reporting symptoms of burnout, including emotional exhaustion [1]. According to the Medscape National Physician Burnout and Suicide Report, the burnout rate among physicians was reported to be as high as 42% in 2020, compared with 39% in 2013 [2,3]. Similarly, high rates were reported for residents with scores between 41% and 74% across different specialties [4]. Furthermore, 27% of residents stated that they rarely or never had time to lead a satisfying social life; of these, 68% reported having failed relationships for this reason [5,6].

During the COVID-19 pandemic, these figures increased further with about 60% of physicians in Canada reporting that their mental health worsened after the pandemic [7]. Similarly, 35% of a sample of emergency residents demonstrated symptoms of posttraumatic stress disorder acutely during the COVID-19 pandemic crisis [8].

The impact of burnout on the physician workforce is substantial, including a higher likelihood for medical errors, lower quality of care, higher costs, and overall worse outcomes along with reduced patient satisfaction [9-12]. Physicians reporting burnout symptoms work for fewer hours and leave clinical medicine at a higher rate than those not affected, and the resultant decreased productivity may exacerbate physician shortfall [13-15]. In addition, physicians with high levels of distress and burnout are more prone to self-perceived or reportedly commit medical errors [4,16,17]. Furthermore, the risk of depression, stress, alcohol use, and suicidality were increasingly reported among postgraduate trainees who have experienced more working hours, burnout, and less sleep time [4,17,18]. On the other hand, physicians who have high empathy and more positive attitudes tend to demonstrate positive attributes with better clinical outcome profiles in terms of developing better doctor-patient relationships associated with a high patient satisfaction and moral reasoning with clinical competence [19-21]. A recent study investigated the strength and significance of the associations of health workforce with multiple health outcomes and COVID-19 excess deaths across countries, using the latest World Health Organization dataset [22]. The study reported that a higher aggregate density of health human resources, particularly physicians, was significantly associated with healthy life expectancy at birth [22].

Addressing stress and burnout among physicians, postgraduate trainees, and medical students is a priority, given that decreasing rates of medical professionals usually seek psychological help or support. In a study among university hospital physicians in Europe, it was reported that overall, more than 3 in 4 distressed physicians had never sought professional help for depression or burnout [23]. In another study that evaluated the help-seeking behaviors of medical students with burnout and compared their stigma perceptions with those of the general US population and age-matched individuals, it was reported that only a third of respondents with burnout (154/454, 33.9%) sought help for an emotional or mental health problem in the last 12 months [24]. Furthermore, medical students with burnout were more likely than those without burnout to agree or strongly agree with 8 of 10 perceived stigma items [24]. It is well-recognized that the stigma and potential professional reputational damage associated with seeking help for mental health problems often deter many physicians, residents, and medical students from seeking psychological support when they are stressed, burnt out, or dealing with anxiety or depression.

Thus, there was a need for an evidence-based, technology-enabled, cost-effective, and geographic location–independent mental health service to address the psychological problems faced by physicians, postgraduate trainees, and medical students. Previous research examining supportive SMS text messages has demonstrated positive outcomes, in terms of improved clinical symptoms and high user satisfaction [25-27]. In a rigorous evaluation of Text4Mood, an SMS text messaging service launched in Fort McMurray, Alberta to address unmet psychological treatment gaps, it was reported that supportive SMS text messages helped the majority of the subscribers feel more hopeful about managing issues (82%), in charge of managing their depression and anxiety (77%), and connected to a support system (75%). Furthermore, a majority reported that the intervention improved their overall mental well-being (83%) [26]. Similar findings were observed in other contextual studies, such as Text4Baby and quit4baby; the 2 programs that have sought to assist women by providing supportive and informative SMS text messaging during pregnancy [28,29]. Participants in such services indicated that receiving these messages gave them a sense of reassurance and made mothers feel supported, particularly in disadvantaged communities [30]. Similarly, texting services, such as Text4Support, have effectively reduced the risk of harm to self and other harm symptoms after 6 months of intervention in a randomized controlled trial [31] and reduced distress, anxiety, and depression symptoms in clinical samples [32]. In addition, the Text4PTSI program has achieved fidelity and significantly reduced psychological distress among public safety personnel post intervention [33,34]. Furthermore, during the COVID-19 pandemic, people needed to feel connected to a health support system. Text4Hope service was provided to support the mental health of the general public in Alberta; the service reported a significant reduction in stress, anxiety, depression symptoms, and suicidal ideation during the pandemic [35-38]. Another related service, the Text4Hope-Addiction program was introduced to people living with substance use disorders [39]. The service reported significant improvement in standardized measures for craving, anxiety, and depression in subscribers. SMS text message–based population-level messaging programs have reported user satisfaction rates of well over 80%, and most subscribers have reported feeling connected to support systems and improving their ability to manage anxiety, depression, and general life issues, suggesting an improvement in mental health literacy [26,27,40-42].

In this study, we propose the Wellness4MDs (Global Psychological e-Health Foundation) initiative, a text-based proposed program that will deliver daily supportive and informative SMS text messages with or without embedded web links for mental health support or literacy resources to the physicians in Canada for 6 months. The Wellness4MDs program SMS text messages are based on cognitive behavioral therapy (CBT) principles and developed by psychiatrists, mental health therapists, clinical psychologists, and users of mental health services. Wellness4MDs program may help provide a first-line intervention that addresses the help-seeking barrier of stigma that fits with the busy lifestyle of physicians, postgraduate trainees, and medical students.

The goal of this project is to support the mental health of physicians, postgraduate trainees, and medical students in Canada using a daily supportive SMS text message. We aim, first, to evaluate the prevalence and correlates of burnout and probable mental health symptoms and, second, to determine if daily supportive SMS text messages can help reduce the prevalence and severity of these symptoms and improve well-being among subscribers in Canada. We will also assess the experience and satisfaction of the Wellness4MDs program subscribers with the daily supportive SMS text messaging program.

## Methods

### Study Design

This is a quantitative longitudinal study with data collected from subscribers of the Wellness4MDs program using a web-based survey at the onset of the program and at designated follow-up timepoints. The surveys will include questions related to sociodemographic characteristics, roles, years of training, specialty, work, and clinical information. The Wellness4MDs program will deliver daily supportive and informative SMS text messages with or without embedded web links for mental health support or literacy resources to subscriber cell phones for 6 months. Participation in the surveys would be voluntary, and nonparticipation in any of the surveys will not impact program participation or service delivery. The Wellness4MDs is a national collaborative project that will leverage stakeholder partnerships to disseminate the program to physicians, postgraduate trainees, and medical students. Potential collaborating organizations include the Royal College of Physicians and Surgeons of Canada, faculties of medicine across Canada, provincial medical associations, provincial health authorities, and provincial medical licensing colleges. Initially, promotional material will be provided and give an overview of the Wellness4MDs program and information about how physicians, postgraduate medical trainees, and medical students can subscribe and unsubscribe to the program. Collaborating organizations will be requested to share information about the Wellness4MDs program with physicians, postgraduate medical trainees, and medical students on their databases and social medical handles, including Facebook, LinkedIn, Instagram, and Twitter (rebranded as X). Thus, the Wellness4MDs recruitment plan will mirror the successful recruitment plan of similar programs in Alberta, including the Text4Hope program that was launched in response to COVID-19 in March 2020 [36,37,43] as a collaborative initiative between the University of Alberta, Alberta Health Services, and six health foundations. Text4Hope was the subject of a wide-exposure communications campaign (television, radio, internet, and print media), including the local provincial mental health foundation, Alberta Health Services, and a media release by the provincial chief medical officer [44].

### Study Setting and Participants

The bilingual Wellness4MDs program will enroll physicians, postgraduate trainees, and medical students in all provinces and territories in Canada. In 2021, the total number of physicians in Canada was 108,040, according to the annual census [45]. According to the Canadian Medical Association, the total number of residents in all postgraduate programs in 2018-2019 was 16,508, and the total enrollment in undergraduate medical schools in Canada in 2017-2018 was 11,737 [46].

### Program Timelines

The Wellness4MDs initiative will be a 3-year project, with timelines as detailed in Table 1.

**Table 1:** Wellness4MDs project Gantt chart.

Milestones			Year 1									Year 2									Year 3						
			Q1^a^		Q2^b^		Q3^c^		Q4^d^		Q1			Q2		Q3		Q4		Q1			Q2		Q3		Q4
**Milestone 1: recruitment of a project staff and creation of the bank of supportive SMS text messages**																											
	1.1 A project coordinator and evaluation specialist would be recruited to support project coordination, implementation, monitoring, and evaluation	✓																									
	1.2 Review and adaptation of the bank of supportive SMS text messages and creation of new messages for the Wellness4MDs program by an expert group	✓																									
**Milestone 2: launch of the Wellness4MDs program, advertisement, and delivery of service**																											
	2.1 Recruitment			✓		✓		✓		✓			✓		✓		✓		✓			✓					
	2.2 Provision of the service			✓		✓		✓		✓			✓		✓		✓		✓			✓		✓		✓	
**Milestone 3: evaluation of the service, including clinical assessment and satisfaction of the participants, and stakeholders’ feedbacks**																											
	3.1 Baseline survey (excluding satisfaction survey)			✓		✓		✓		✓			✓		✓		✓		✓			✓		✓		✓	
	3.2 Follow-up survey (including satisfaction survey)					✓		✓		✓			✓		✓		✓		✓			✓		✓		✓	
	3.3 Key informant interviews and Focus group workshops												✓									✓					
**Milestone 4: data compilation, analysis, and preparation of reports, publications, and presentations for multi-scale dissemination**																											
	4.1 Data compilation							✓		✓			✓		✓		✓		✓			✓		✓		✓	
	4.2 Data analysis							✓		✓			✓		✓		✓		✓			✓		✓		✓	
	4.3 Preparation of reports, publications, and presentations							✓		✓			✓		✓		✓		✓			✓		✓		✓	

^a^Q1: first quarter.

^b^Q2: second quarter.

^c^Q3: third quarter.

^d^Q4: fourth quarter.

### Wellness4MDs Program

The Wellness4MDs program is modeled on the highly successful Text4Hope and Wellness4Teachers initiatives in Canada [35,47]. The program is powered by the ResilienceNHope software, a web-based application that delivers the suite of supportive SMS text and email messaging programs, including Text4Mood, Text4Hope, Wellness4Teachers, Text4PTSI, Text4Wellness, and MoreGoodDays [48]. Canadian physicians, postgraduate trainees, and medical students will be able to subscribe to the Wellness4MDs program by texting “WellMD” to a designated phone number. Individuals can unsubscribe to the program by simply texting “stop” to the program phone number. The Wellness4MDs program will deliver daily supportive and informative SMS text messages (some with embedded web links for mental health support or literacy resources) to subscriber cell phones for 6 months. The Wellness4MDs program SMS text messages are based on CBT principles and were developed by psychiatrists, mental health therapists, clinical psychologists, and users of mental health services. About a third of the SMS text messages will have embedded web links that include additional physician health and wellness resources from reputable sources.

### Examples of SMS Text Messages

The following are example SMS text messages that will be sent.

1. “Perfectionism can cause anxiety. Remember a perfect work environment does not exist. Try deliberately being imperfect and see what happens. Do your anxious thoughts come true? Remember that there are many possibilities in life. Try not to limit yourself from enjoying all that is possible.” In addition, an embedded web link has been provided [49].
2. “We each have different signature strengths. It’s not about competing with others but learning our unique strengths. Strengths come in many varieties, such as humor, curiosity, persistence, kindness, love, forgiveness, cooperativeness, and optimism. Share your strength with others. Help a struggling colleague.”
3. “Problems are almost never solved in one step. See if you can break the problem into steps. Take the first step today by starting with a manageable situation. Get the support of a friend, family, colleagues, or supervisor.” In addition, an embedded web link has been provided [50].

### Hypothesis

We hypothesize that the Wellness4MDs program will achieve (1) at least a 20% reduction in mean scores for burnout, anxiety, and depressive symptoms on validated scales and a 20% increase in self-reported well-being at 6 weeks, 3 months, and 6 months after receiving the service, and (2) at least 80% of subscribers will express satisfaction with the program and perceive the daily supportive SMS text messages as contributing to their overall mental well-being.

### Sample Size Calculation

With a projection that the effect size for the reduction in mean 7-item Generalized Anxiety Disorder (GAD-7) scale and 9-item Patient Health Questionnaire (PHQ-9) scores at 6 months from baseline would be 0.1, a population variance of 1 for each scale mean score, a 2-sided significance level α=.05, and a power of 90% (β=.1), using a web-based script [51], we estimated that the sample size needed to assess the effects of the daily supportive SMS text messages on the outcome variables would be 1053.

### Data Collection and Outcome Measures

The project will run for 3 consecutive years. Quantitative data will be collected from subscribers of the Wellness4MDs program using a web-based survey at the onset of the program (baseline), 6 weeks, 3 months (midpoint), and 6 months (end of service) as illustrated in Table 2. The surveys will include demographic questions such as sex and gender, as well as information about their role (physicians, postgraduate trainees, or medical students), their years of training or practice, and their specialty of retaining or practice if they are residents or physicians, in addition to the clinical outcome measures.

Primary outcome measures include changes in the mean scores on the PHQ-9 [51], the GAD-7 [52], the Maslach Burnout Inventory (MBI) [53], and the World Health Organization-Five Well-Being Index (WHO-5) [54] for subscribers from baseline to 6 weeks, 3 months, and 6 months.

The PHQ-9 is a self-administered scale designed to measure likely depression. It is an effective tool for patients and a validated tool for screening of depressive symptoms among the general population [55]. Symptoms experienced by patients during the 2 weeks before answering questionnaires are considered. Scores range from never (0), several days (1), more than half of days (2), to 3 (nearly every day), with every item ranging from 0 to 27 [51,56]. The tool’s reliability and validity have shown good psychometric properties with accepted sensitivity using 10 or above as a cutoff score and high internal consistency (Cronbach α=0.79) [55,57].

The GAD-7 scale is a self-administered valid tool designed to measure likely anxiety [58,59]. The 7 self-reported items are used to assess the severity of generalized anxiety disorder (GAD) symptoms over the past 2 weeks. Ratings are based on a 4-point Likert scale: not at all sure (0), several days (1), over half the days (2), and nearly every day (3). The scores ranged from 0 to 21, with higher scores indicative of more severe symptoms. A sensitivity of 89% and a specificity of 82% for a cutoff score of 10 [59] are considered. The GAD-7 also showed good test-retest reliability (intraclass correlation 0.83), as well as excellent internal consistency (Cronbach α=0.92) [58,60].

The WHO-5 is a self-reported measure of subjective well-being that is highly useful in clinical practice and in research studies to assess well-being over time or to compare well-being between groups [61]. The scale includes 5 positively worded items with a 6-point Likert scale used to assess participant’s feelings in the last 2 weeks, ranging from 0 (not present) to 5 (constantly present). The raw scores are transformed into a score from 0 (worst thinkable well-being) to 100 (best thinkable well-being) [62]. Having a score <50 indicates poor emotional well-being and requires further evaluation. The WHO-5 demonstrated satisfactory internal consistency reliability and concurrent validity with other scales [61,62].

**Table 2:** Client-oriented outcome measures and feedbacks and experiences of clients and stakeholders.

Construct	Tool (scale)	T_0_^a^	T_1_^b^	T_2_^c^	T_3_^d^
Likely MDD^e^	PHQ-9^f^	✓	✓	✓	✓
Likely GAD^g^	GAD-7^h^	✓	✓	✓	✓
Burnout	Maslach Burnout Inventory	✓	✓	✓	✓
Mental well-being	WHO-5^i^	✓	✓	✓	✓
Satisfaction with the service	Standardized satisfaction survey		✓	✓	✓
Experience and feedback	Focus groups and key informant interviews				✓

^a^T_0_: baseline timepoint.

^b^T_1_: 6-week timepoint.

^c^T_2_: 3-month timepoint.

^d^T_3_: 6-month timepoint.

^e^MDD: major depressive disorder.

^f^PHQ-9: 9-item Patient Health Questionnaire.

^g^GAD: generalized anxiety disorder.

^h^GAD-7: 7-item Generalized Anxiety Disorder.

^i^WHO-5: World Health Organization-Five Well-Being Index.

The MBI, a short questionnaire-based tool, is designed to measure the symptoms and severity of burnout. In total, 3 main domains are examined, that are depersonalization, emotional exhaustion, and professional fulfillment index. The professional fulfillment index includes 2 domains, that are work exhaustion and interpersonal disengagement (Burnout scale) and the professional fulfillment scale. The reliability of MBI is supported by several studies, where Cronbach α ratings of 0.90 for emotional exhaustion, 0.76 for depersonalization, and 0.76 for personal accomplishment [63].

Secondary outcome measures include changes in prevalence in subscribers of burnout, likely GAD and likely major depressive disorder (MDD), from baseline to 6 weeks, 3 months, and 6 months measured using the GAD-7, the PHQ-9, and the MBI. Other secondary outcome measures include subscriber satisfaction.

### Statistical Analysis

Quantitative data will be analyzed with descriptive statistics, paired *t* test, and chi-square tests using SPSS Statistics for Windows (Version 25; IBM Corp) [64]. Descriptive data will be presented as numbers and percentages. Paired *t* test will be used to assess the differences in the mean scores of each of the outcome scales for the baseline and the follow-up time points. In addition, the chi-square test will be used to compare the prevalence of burnout, likely GAD and likely MDD, from baseline to the follow-up time points.

### Ethical Considerations

This study has been approved by the human ethics review boards of the University of Alberta (Pro00129541). Informed consent will be implied if subscribers access the study information leaflet, complete the survey questions, and submit responses. Study data will be anonymized and deidentified using encrypted files that will be accessible only to the principal investigator and the research coordinator. No identification of individual participants in any images of the paper or supplementary material. No compensation will be used. No incentives will be provided to the study participants.

## Results

The project was launched in the fourth quarter of 2023, and program evaluation results will become available by January 2027. Aligned with similar previous initiatives, the Wellness4MDs program is expected to reduce the prevalence and severity of psychological problems among physicians in Canada and achieve high subscriber satisfaction.

## Discussion

### Principal Findings

The Wellness4MDs project represents an innovative initiative that aligns with 2 main strategic directions, namely, highest quality and proactive and innovative approach. In terms of highest quality, the evidence in favor of the potential effectiveness of the Wellness4MDs program to alleviate stress, burnout, anxiety, and depression among physicians, postgraduate medical trainees, and medical students is derived from meta-analyses of randomized controlled trials and evaluations of related population-level interventions [26,31,32,36-38,40,65-67]. For example, in 2 randomized controlled trials, when CBT-based daily supportive SMS text messages were delivered to patients with MDD either as part of inpatient postdischarge community follow-up care or as part of outpatient clinic care, there was a 50% and 25% greater reduction in mean depression symptoms scores measured on the Beck’s Depression Inventory Scale compared with the control group, respectively [68,69]. Similarly, in an evaluation of the Text4Hope program launched in Alberta during the COVID-19 pandemic, subscribers who had received daily supportive SMS text messages for just 6 weeks had a significantly lower prevalence of moderate to high stress, likely generalized anxiety disorder, likely MDD, suicidal ideation, and disturbed sleep compared with new subscribers during the same time frame [37]. The Wellness4MDs program therefore has the potential to offer physicians an intervention that is of the highest quality with respect to the evidence of effectiveness. The approach to using daily supportive and informative SMS text messages to promote physician wellness would be innovative as it will be the first of its kind globally. In addition to addressing issues of stress, burnout, anxiety, and depression among physicians, residents, and medical students, the embedded web links in some of the messages to be preprogrammed into the Wellness4MDs program are expected to improve the mental health literacy of subscribers and provide information on complimentary mental health resources and approaches that would also be proactive and innovative.

The goal of this project is to provide mental health support and information about available resources to physicians, postgraduate medical trainees, and medical students in Canada through a daily supportive SMS text message program. Similar to the impressive uptake by teachers in Canada of the Wellness4Teachers program (close to 2000 teachers subscribed within 3 months) [47], we anticipate high subscriptions to the Wellness4MDs program from physicians, postgraduate medical trainees, and medical students across Canada. Furthermore, similar to outcomes achieved from the Text4Hope program launched for the general public during the COVID-19 pandemic, we anticipate that the Wellness4MDs program will reduce stress, burnout, anxiety, and depression among physicians, postgraduate medical trainees, and medical students. The expected long-term outcomes could include lower rates of absenteeism due to stress and burnout, reduced physician turnover, reduced medical errors in clinical practice, better safety profile of patients and patient satisfaction, and fewer patient complaints. Thus, we anticipate the benefits of the Wellness4MDs program will lead to better health profiles for physicians, postgraduate medical trainees, and medical students, and the health care system will ultimately benefit through cost savings from the avoidance of expenses that directly relate to or stem from these activities.

### Limitations of the Study

This study has a number of limitations. First, the self-reported scales to be used for assessment of the psychological problem, although validated, are not diagnostic. In addition, it is highly probable that the physicians, postgraduate medical trainees, and medical students who are impacted by burnout, stress, or anxiety may be more likely to subscribe to the Wellness4MDs program and so be overrepresented in this study, and thus impact the generalizability of the prevalence estimated for psychological problems. Finally, the duration of the intervention is limited for 6 months; thus, the long-term outcomes after cessation of the service will not be established. These limitations notwithstanding, this study is the first to provide e–mental health support for stress, burnout, anxiety, and depression among physicians, postgraduate medical trainees, and medical students in Canada. This study is a pioneer in assessing if daily supportive SMS text messages delivered through the Wellness4MDs program can reduce the prevalence and severity of stress, burnout, anxiety, and depression, and improve well-being among physicians, postgraduate medical trainees, and medical students. The findings would therefore be of interest to policy makers, especially those working in the health care sector.

### Conclusion

The Wellness4MDs program is an innovative first-line population-level, cost-effective, and easily scalable e–mental health program that is expected to reduce psychological problems in physicians, postgraduate medical trainees, and medical students in Canada. The successful implementation of Wellness4MDs and positive outcomes will provide the imperative and overarching guidance on the design, implementation, and evaluation of similar e–mental health interventions tailored for the health workforce globally, including nurses and allied health professionals.

## Abbreviations

CBT: cognitive behavioral therapy
GAD: generalized anxiety disorder
GAD-7: 7-item Generalized Anxiety Disorder
MBI: Maslach Burnout Inventory
MDD: major depressive disorder
PHQ-9: 9-item Patient Health Questionnaire
WHO-5: World Health Organization–Five Well-Being Index

## Data Availability

The datasets generated or analyzed during this study are available from the corresponding author upon reasonable request.

## References

[1] (2022). National physician health survey. Canadian Medical Association.

[2] (2020). Medscape National Physician Burnout & Suicide Report.

[3] De Hert S (2020). Burnout in healthcare workers: prevalence, impact and preventative strategies. Local Reg Anesth.

[4] Ames SE, Cowan JB, Kenter K, Emery S, Halsey D (2017). Burnout in orthopaedic surgeons: a challenge for leaders, learners, and colleagues: aoa critical issues. J Bone Joint Surg Am.

[5] (2020). Medscape Residents Lifestyle & Happiness Report.

[6] (2021). Medscape.

[7] Canadian physicians report declines in mental health, professional fulfillment. Medscape.

[8] Chang J, Ray J, Joseph D, Evans L, Joseph M (2022). Burnout and post-traumatic stress disorder symptoms among emergency medicine resident physicians during the COVID-19 pandemic. West J Emerg Med.

[9] Tawfik DS, Profit J, Morgenthaler TI, Satele DV, Sinsky CA, Dyrbye LN, Tutty MA, West CP, Shanafelt TD (2018). Physician burnout, well-being, and work unit safety grades in relationship to reported medical errors. Mayo Clin Proc.

[10] (2019). Medscape.

[11] Patel RS, Bachu R, Adikey A, Malik M, Shah M (2018). Factors related to physician burnout and its consequences: a review. Behav Sci (Basel).

[12] Panagioti M, Geraghty K, Johnson J, Zhou A, Panagopoulou E, Chew-Graham C, Peters D, Hodkinson A, Riley R, Esmail A (2018). Association between physician burnout and patient safety, professionalism, and patient satisfaction: a systematic review and meta-analysis. JAMA Intern Med.

[13] Shanafelt TD, Dyrbye LN, West CP, Sinsky CA (2016). potential impact of burnout on the US physician workforce. Mayo Clin Proc.

[14] Shanafelt TD, Mungo M, Schmitgen J, Storz KA, Reeves D, Hayes SN, Sloan JA, Swensen SJ, Buskirk SJ (2016). Longitudinal study evaluating the association between physician burnout and changes in professional work effort. Mayo Clin Proc.

[15] Willard-Grace R, Knox M, Huang B, Hammer H, Kivlahan C, Grumbach K (2019). Burnout and health care workforce turnover. Ann Fam Med.

[16] Romani M, Ashkar K (2014). Burnout among physicians. Libyan J Med.

[17] Kwok C (2021). Depression, stress, and perceived medical errors in singapore psychiatry residents. Acad Psychiatry.

[18] Dyrbye LN, Massie FS, Eacker A, Harper W, Power D, Durning SJ, Thomas MR, Moutier C, Satele D, Sloan J, Shanafelt TD (2010). Relationship between burnout and professional conduct and attitudes among US medical students. JAMA.

[19] Levinson W, Roter D (1995). Physicians' psychosocial beliefs correlate with their patient communication skills. J Gen Intern Med.

[20] Suchman AL, Roter D, Green M, Lipkin M (1993). Physician satisfaction with primary care office visits. Collaborative study group of the American academy on physician and patient. Med Care.

[21] Hojat M, Louis DZ, Markham FW, Wender R, Rabinowitz C, Gonnella JS (2011). Physicians' empathy and clinical outcomes for diabetic patients. Acad Med.

[22] Liu J, Eggleston K (2022). The association between health workforce and health outcomes: a cross-country econometric study. Soc Indic Res.

[23] Fridner A, Belkić K, Marini M, Gustafsson Sendén M, Schenck-Gustafsson K (2012). Why don't academic physicians seek needed professional help for psychological distress?. Swiss Med Wkly.

[24] Dyrbye LN, Eacker A, Durning SJ, Brazeau C, Moutier C, Massie FS, Satele D, Sloan JA, Shanafelt TD (2015). The impact of stigma and personal experiences on the help-seeking behaviors of medical students with burnout. Acad Med.

[25] Agyapong VI, McLoughlin DM, Farren CK (2013). Six-months outcomes of a randomised trial of supportive text messaging for depression and comorbid alcohol use disorder. J Affect Disord.

[26] Agyapong V, Mrklas K, Juhás M, Omeje J, Ohinmaa A, Dursun SM, Greenshaw AJ (2016). Cross-sectional survey evaluating Text4Mood: mobile health program to reduce psychological treatment gap in mental healthcare in Alberta through daily supportive text messages. BMC Psychiatry.

[27] Agyapong VIO, Milnes J, McLoughlin DM, Farren CK (2013). Perception of patients with alcohol use disorder and comorbid depression about the usefulness of supportive text messages. Technol Health Care.

[28] Evans WD, Wallace Bihm J, Szekely D, Nielsen P, Murray E, Abroms L, Snider J (2014). Initial outcomes from a 4-week follow-up study of the Text4baby program in the military women's population: randomized controlled trial. J Med Internet Res.

[29] Abroms LC, Johnson PR, Heminger CL, Van Alstyne JM, Leavitt LE, Schindler-Ruwisch JM, Bushar JA (2015). Quit4baby: results from a pilot test of a mobile smoking cessation program for pregnant women. JMIR Mhealth Uhealth.

[30] Chandra PS, Sowmya H, Mehrotra S, Duggal M (2014). 'SMS' for mental health—feasibility and acceptability of using text messages for mental health promotion among young women from urban low income settings in India. Asian J Psychiatr.

[31] Shalaby R, Spurvey P, Knox M, Rathwell R, Vuong W, Surood S, Urichuk L, Snaterse M, Greenshaw AJ, Li X, Agyapong VIO (2022). Clinical outcomes in routine evaluation measures for patients discharged from acute psychiatric care: four-arm peer and text messaging support controlled observational study. Int J Environ Res Public Health.

[32] Noble JM, Vuong W, Surood S, Urichuk L, Greenshaw AJ, Agyapong VIO (2021). Text4Support mobile-based programming for individuals accessing addictions and mental health services-retroactive program analysis at baseline, 12 weeks, and 6 months. Front Psychiatry.

[33] Obuobi-Donkor G, Eboreime E, Bond J, Phung N, Eyben S, Hayward J, Zhang Y, MacMaster F, Clelland S, Greiner R, Jones C, Cao B, Brémault-Phillips S, Wells K, Li X, Hilario C, Greenshaw AJ, Agyapong VIO (2022). An E-mental health solution to prevent and manage posttraumatic stress injuries among first responders in alberta: protocol for the implementation and evaluation of text messaging services (Text4PTSI and Text4Wellbeing). JMIR Res Protoc.

[34] Obuobi-Donkor G, Shalaby R, Eboreime E, Agyapong B, Phung N, Eyben S, Wells K, Hilario C, Dias RDL, Jones C, Brémault-Phillips S, Zhang Y, Greenshaw AJ, Agyapong VIO (2023). Text4PTSI: a promising supportive text messaging program to mitigate psychological symptoms in public safety personnel. Int J Environ Res Public Health.

[35] Agyapong VIO, Hrabok M, Vuong W, Gusnowski A, Shalaby R, Mrklas K, Li D, Urichuk L, Snaterse M, Surood S, Cao B, Li X, Greiner R, Greenshaw AJ (2020). Closing the psychological treatment gap during the COVID-19 pandemic with a supportive text messaging program: protocol for implementation and evaluation. JMIR Res Protoc.

[36] Agyapong VIO, Hrabok M, Vuong W, Shalaby R, Noble JM, Gusnowski A, Mrklas KJ, Li D, Urichuk L, Snaterse M, Surood S, Cao B, Li X, Greiner R, Greenshaw AJ (2020). Changes in stress, anxiety, and depression levels of subscribers to a daily supportive text message program (Text4Hope) during the COVID-19 pandemic: cross-sectional survey study. JMIR Ment Health.

[37] Agyapong VIO, Shalaby R, Hrabok M, Vuong W, Noble JM, Gusnowski A, Mrklas K, Li D, Snaterse M, Surood S, Cao B, Li X, Greiner R, Greenshaw AJ (2021). Mental health outreach via supportive text messages during the COVID-19 pandemic: improved mental health and reduced suicidal ideation after six weeks in subscribers of Text4Hope compared to a control population. Int J Environ Res Public Health.

[38] Agyapong VIO, Hrabok M, Shalaby R, Vuong W, Noble JM, Gusnowski A, Mrklas K, Li D, Urichuck L, Snaterse M, Surood S, Cao B, Li X, Greiner R, Greenshaw AJ (2022). Text4Hope: receiving daily supportive text messages for 3 months during the COVID-19 pandemic reduces stress, anxiety, and depression. Disaster Med Public Health Prep.

[39] Obuobi-Donkor G, Shalaby R, Vuong W, Agyapong B, Hrabok M, Gusnowski A, Surood S, Greenshaw AJ, Agyapong VI (2023). Effects of Text4Hope-addiction support program on cravings and mental health symptoms: results of a longitudinal cross-sectional study. JMIR Form Res.

[40] Shalaby R, Vuong W, Hrabok M, Gusnowski A, Mrklas K, Li D, Snaterse M, Surood S, Cao B, Li X, Greiner R, Greenshaw AJ, Agyapong VIO (2021). Gender differences in satisfaction with a text messaging program (Text4Hope) and anticipated receptivity to technology-based health support during the COVID-19 pandemic: cross-sectional survey study. JMIR Mhealth Uhealth.

[41] Shalaby R, Adu MK, El Gindi HM, Agyapong VIO (2022). Text messages in the field of mental health: rapid review of the reviews. Front Psychiatry.

[42] Shalaby R, Vuong W, Hrabok M, Gusnowski A, Mrklas K, Li D, Snaterse M, Surood S, Cao B, Li X, Greiner R, Greenshaw AJ, Agyapong VIO (2021). Gender differences in satisfaction with a text messaging program (Text4Hope) and anticipated receptivity to technology-based health support during the COVID-19 pandemic: cross-sectional survey study. JMIR Mhealth Uhealth.

[43] Agyapong VIO (2020). Coronavirus disease 2019 pandemic: health system and community response to a text message (Text4Hope) program supporting mental health in Alberta. Disaster Med Public Health Prep.

[44] Braid T (2020). You are NOT alone! Text4Hope aims to help Albertans shoot down the Covid-19 Blues. Todayville Edmonton.

[45] (2021). 2021 Census of population key indicators by geography: Canada. Statistics Canada.

[46] Quick facts on Canada's physicians. Canadian Medical Association.

[47] Agyapong B, Wei Y, da Luz Dias R, Agyapong VIO (2022). Burnout and associated psychological problems among teachers and the impact of the Wellness4Teachers supportive text messaging program: protocol for a cross-sectional and program evaluation study. JMIR Res Protoc.

[48] Email and text message programs that support mental health. Resilience N Hope.

[49] 10 ways to overcome perfectionism. Oregon Counseling.

[50] Solving problems. Course Hero.

[51] Kroenke K, Spitzer RL, Williams JBW (2001). The PHQ-9: validity of a brief depression severity measure. J Gen Intern Med.

[52] Spitzer RL, Kroenke K, Williams JBW, Löwe B (2006). A brief measure for assessing generalized anxiety disorder: the GAD-7. Arch Intern Med.

[53] Maslach CJS, Leiter M (1996). The Maslach Burnout Inventory Manual.

[54] The World Health Organisation- Five Well-Being Index (WHO-5). Child Outcome Research Consortium.

[55] Shin C, Ko Y, An H, Yoon H, Han C (2020). Normative data and psychometric properties of the patient health questionnaire-9 in a nationally representative Korean population. BMC Psychiatry.

[56] Mao W, Adu M, Eboreime E, Shalaby R, Nkire N, Agyapong B, Pazderka H, Obuobi-Donkor G, Owusu E, Oluwasina F, Zhang Y, Agyapong VIO (2022). Post-traumatic stress disorder, major depressive disorder, and wildfires: a fifth-year postdisaster evaluation among residents of fort McMurray. Int J Environ Res Public Health.

[57] Levis B, Benedetti A, Thombs BD, DEPRESsion Screening Data (DEPRESSD) Collaboration (2019). Accuracy of patient health questionnaire-9 (PHQ-9) for screening to detect major depression: individual participant data meta-analysis. BMJ.

[58] Kroenke K, Spitzer RL, Williams JB, Monahan PO, Löwe B (2007). Anxiety disorders in primary care: prevalence, impairment, comorbidity, and detection. Ann Intern Med.

[59] Williams N (2014). The GAD-7 questionnaire. Occup Med.

[60] Owusu E, Shalaby R, Eboreime E, Nkire N, Agyapong B, Obuobi-Donkor G, Adu MK, Mao W, Oluwasina F, Lawal MA, Agyapong VIO (2022). Prevalence and predictors of generalized anxiety disorder symptoms in residents of fort McMurray five years after the devastating wildfires. Trauma Care.

[61] Topp CW, Østergaard SD, Søndergaard S, Bech P (2015). The WHO-5 Well-Being Index: a systematic review of the literature. Psychother Psychosom.

[62] de Wit M, Pouwer F, Gemke RJBJ, Delemarre-van de Waal HA, Snoek FJ (2007). Validation of the WHO-5 Well-Being Index in adolescents with type 1 diabetes. Diabetes Care.

[63] Maslach Burnout Inventory (MBI). Complete Dissertaion by Statistics Solutions.

[64] Release notes - IBM® SPSS® Statistics 25.0. IBM.

[65] Senanayake B, Wickramasinghe SI, Chatfield MD, Hansen J, Edirippulige S, Smith AC (2019). Effectiveness of text messaging interventions for the management of depression: a systematic review and meta-analysis. J Telemed Telecare.

[66] Shalaby R, Agyapong B, Vuong W, Hrabok M, Gusnowski A, Surood S, Greenshaw AJ, Agyapong VIO (2022). Naturalistic randomized controlled trial demonstrating effectiveness of Text4Hope in supporting male population mental health during the COVID-19 pandemic. Front Public Health.

[67] Shalaby R, Hrabok M, Spurvey P, Abou El-Magd RM, Knox M, Rude R, Vuong W, Surood S, Urichuk L, Snaterse M, Greenshaw AJ, Li X, Agyapong VIO (2021). Recovery following peer and text messaging support after discharge from acute psychiatric care in Edmonton, Alberta: controlled observational study. JMIR Form Res.

[68] Agyapong VI, Ahern S, McLoughlin DM, Farren CK (2012). Supportive text messaging for depression and comorbid alcohol use disorder: single-blind randomised trial. J Affect Disord.

[69] Agyapong VIO, Juhás M, Ohinmaa A, Omeje J, Mrklas K, Suen VYM, Dursun SM, Greenshaw AJ (2017). Randomized controlled pilot trial of supportive text messages for patients with depression. BMC Psychiatry.

